# Supplementary material for: Integrated NMR and MS Analysis of the Plasma Metabolome Reveals Major Changes in One-Carbon, Lipid, and Amino Acid Metabolism in Severe and Fatal Cases of COVID-19
Source: Metabolites. 2023 Jul 24;13(7):879. doi: 10.3390/metabo13070879 (PMC10384698; doi:10.3390/metabo13070879)
Supplement: Supplementary file 1 [file metabolites-13-00879-s001.zip › metabolites-2473658-supplementary.pdf]

Supplementary Material

Integrated NMR and MS Analysis of the Plasma Metabolome Reveals Major Changes in One-Carbon, Lipid, and Amino Acid Metabolism in Severe and Fatal Cases of COVID-19

Marcos C. Gama-Almeida [1,†], Gabriela D. A. Pinto [1,†], Lívia Teixeira [2], Eugenio D. Hottz [3], Paula Ivens [4], Hygor Ribeiro [4,5], Rafael Garrett [4], Alexandre G. Torres [1,5], Talita I. A. Carneiro [1], Bianca de O. Barbalho [1], Christian Ludwig [6], Claudio J. Struchiner [7,8], Iranaia Assunção-Miranda [9], Ana Paula C. Valente [10], Fernando A. Bozza [11,12], Patrícia T. Bozza [2], Gilson C. dos Santos, Jr. [13,*] and Tatiana El-Bacha [1,5,*]

1 LeBioME-Bioactives, Mitochondrial and Placental Metabolism Core, Institute of Nutrition Josué de Castro, Universidade Federal do Rio de Janeiro, Rio de Janeiro 21941-902, Brazil; marcosalmeidajj@gmail.com (M.C.G.-A.); gabidap@gmail.com (G.D.A.P.); torres@iq.ufrj.br (A.G.T.); talita.tiac@gmail.com (T.I.A.C.); biancabarbalho@ufrj.br (B.d.O.B.)
2 Laboratory of Immunopharmacology, Oswaldo Cruz Institute, Oswaldo Cruz Foundation, Rio de Janeiro 21041-361, Brazil; liviaimunofar@gmail.com (L.T.); pbozza@ioc.fiocruz.br (P.T.B.)
3 Laboratory of Immunothrombosis, Department of Biochemistry, Federal University of Juiz de Fora, Juiz de Fora 36936-900, Brazil; eugenio.hottz@icb.ufjf.br
4 LabMeta, Metabolomics Laboratory, Institute of Chemistry, Universidade Federal do Rio de Janeiro, Rio de Janeiro 21941-598, Brazil; ivenspaula@gmail.com (P.I.); ribeirohygor@ufrj.br (H.R.); rafael_garrett@iq.ufrj.br (R.G.)
5 Lipid Biochemistry and Lipidomics Laboratory, Department of Chemistry, Universidade Federal do Rio de Janeiro, Rio de Janeiro 21941-598, Brazil
6 Institute of Metabolism and Systems Research, University of Birmingham, Birmingham B15 2SQ, UK; c.ludwig@bham.ac.uk
7 School of Applied Mathematics, Fundação Getúlio Vargas, Rio de Janeiro 22231-080, Brazil; claudio.struchiner@fgv.br
8 Institute of Social Medicine, Universidade do Estado do Rio de Janeiro, Rio de Janeiro 20550-013, Brazil
9 LaRIV, Instituto de Microbiologia Paulo de Goes, Universidade Federal do Rio de Janeiro, Rio de Janeiro 21941-902, Brazil; iranaiamiranda@micro.ufrj.br
10 National Center for Nuclear Magnetic Resonance—Jiri Jonas, Institute of Medical Biochemistry, Universidade Federal do Rio de Janeiro, Rio de Janeiro 21941-902, Brazil; valente@cnrmn.bioqmed.ufrj.br
11 National Institute of Infectious Disease Evandro Chagas, Oswaldo Cruz Foundation, Rio de Janeiro 21040-360, Brazil; fernando.bozza@ini.fiocruz.br
12 D'Or Institute for Research and Education, Rio de Janeiro 22281-100, Brazil
13 LabMet-Laboratory of Metabolomics, Instituto de Biologia Roberto Alcantara Gomes (IBRAG), Department of Genetics, State University of Rio de Janeiro, Rio de Janeiro 20551-030, Brazil
* Correspondence: gilson.junior@uerj.br (G.C.d.S.J.); tatiana@nutricao.ufrj.br (T.E.-B.)
† These authors contributed equally to this work.

Supplementary Table S1: Assignment table of metabolites that discriminated in the PCA and univariate analyses

Supplementary Table S2: Assignment table of broad signals of compounds that discriminated in the PCA and univariate analysis

Supplementary Table S3: Parameters used for untargeted analysis in MS-DIAL software

Supplementary Table S4: List of the target compounds monitored in the method at pH 8

Supplementary Table S5: List of the target compounds monitored in the method at pH 3

Supplementary Table S6: Assigned metabolites with significant differences among groups according to the non-targeted MS-metabolomics in the negative mode.

Supplementary Table S7: Assigned metabolites according to the non-targeted MS-metabolomics in the positive mode.

Supplementary Figure S1. PCA loading factors plot highlights the discriminant metabolites for the separation of groups according to ^{1}H NMR-based metabolomics.

Supplementary Figure S2. PC1 scores plot versus sample in run order at pH 8 and pH 3 analyses indicating the stability of the system throughout the analytical run.

Supplementary Figure S3: Classification and Regression Tree (CART) model indicates that $(CH_3)_3$-choline related metabolites and creatine/creatinine present high predictive power in assigning subjects to their morbidity class.

Supplementary Figure S4: Classification and Regression Tree (CART) model indicates that $(CH_3)_3$-choline related metabolites and *N*-acetylserine present high predictive power in assigning subjects to their morbidity class.

Supplementary Figure S5: Classification and Regression Tree (CART) model indicates that hypoxanthine and *N*-acetylserine present high predictive power in assigning subjects to their morbidity class.

Supplementary Figure S6: Classification and Regression Tree (CART) model indicates that *N*-acetylserine presents high predictive power in assigning subjects to their morbidity class.

Supplementary table S1: Assignment table of metabolites that discriminated in the PCA and univariate analysis

The peak report of assigned compounds can be seen at https://spin.ccic.osu.edu/index.php/colmarm, session ID session ID 3121-pZ5ZukwXBh (COLMAR https://spin.ccic.osu.edu/index.php/colmarm/index2).

Metabolite	Chemical Shift (ppm)
$(CH_3)_3$ choline	3.19
Acetate	1.90
Acetoacetate	2.22
Alanine	1.47
Arginine	1.69
Creatine/Creatinine	3.03
Formate	8.44
Glutamate+Glutamine	2.15
Glutamine	2.44
Glycine	3.55
Histidine	7.11, 8.01
Isoleucine	0.94
Lactate	1.31
Leucine	0.95
Tyrosine	6.87, 7.17
Valine	0.98, 1.03

Supplementary Table S2: Assignment table of broad signals of compounds that discriminated in the PCA and univariate analysis

The peak report of assigned compounds can be seen at https://spin.ccic.osu.edu/index.php/colmarm, session ID session ID 3121-pZ5ZukwXBh (COLMAR https://spin.ccic.osu.edu/index.php/colmarm/index2).

**Lipids and proteins (broad signal)**	**Chemical Shift (ppm)**
$(CH_3)_3$ choline-related signals	3.19
CH_3 of lipoproteins	1.24
CH_2 of lipoproteins	0.85
CH=CH olefinic protons of triacylglycerols	5.28
N-acetyl of glycoproteins	2.04

Supplementary Table S3: Parameters used for untargeted analysis in MS-DIAL software.

**Parameters**	**pH8**	**pH3**
MS1 tolerance	0.005	0.005
MS2 tolerance	0.05	0.05
minimum peak height	5000,000	5000,000
mass slice width	0.05	0.1
sigma window value for deconvolution tolerances for peak alignment	0.5 - Liner weighted moving average	0.5 - Liner weighted moving average
Smoothing method		
Smoothing level	3 scans	3 scans
Minimum peak width	5 scans	5 scans

Supplementary Table S4: List of the target compounds monitored in the method at pH 8.

Nº	Compound name	Rt (min)	Adduct	Molecular formula	Precursor ion	Fragment ion 1	Fragment ion 2	Fragment ion 3
1	Hypoxanthine	5.22	[M-H]-	C5H4N4O	135.03124	92.02576	-	-
2	D-Glucuronic acid	7.23	[M-H]-	C6H10O7	193.03538	113.02451	72.99319	103.00372
3	Taurine	6.72	[M-H]-	C2H7NO3S	124.00738	79.95819	-	-
4	2-Ketoglutaric acid	7.13	[M-H]-	C5H6O5	145.01425	101.02445	128.03522	83.05022
5	*p*-Hydroxyphenyl lactic acid	5.83	[M-H]-	C9H10O4	181.05063	163.04033	135.04541	119.05037
6	Pyroglutamic acid	6.74	[M-H]-	C5H7NO3	128.03532	82.03000	-	-
7	alpha-Hydroxy-isocaproic acid	4.57	[M-H]-	C6H12O3	131.07137	85.06590	113.06087	69.03458
8	D(+)-Glyceric acid	6.78	[M-H]-	C3H6O4	105.01933	72.99324	75.00884	59.01379
9	Malonic acid	7.07	[M-H]-	C3H4O4	103.00368	59.01285	-	-
10	alpha-Lactose/D(+)-Maltose	7.36	[M-H]-	C12H22O11	341.10893	161.04465	101.02317	71.01254
11	L-Isoleucine	6.44	[M+H]+	C6H13NO2	132.10189	86.09673	69.07040	-
12	L-Leucine	6.34	[M+H]+	C6H13NO2	132.10189	86.09673	-	-
13	S-adenosylmethionine	8.28	[M+H]+	C15H22N6O5S	399.14451	-	-	-
14	L-phenylalanine	6.29	[M+H]+	C9H11NO2	166.08625	120.08086	131.04915	149.05968
15	Lactic acid	6.26	[M-H]-	C3H6O3	89.02442	71.01399	-	-
16	Mannose/Galactose/Glucose	6.80	[M-H]-	C6H12O6	179.05611	59.01255	71.01252	89.02314
17	2-Methylbutyrylglycine	5.75	[M-H]-	C7H13NO3	158.08227	74.02499	114.09274	-
18	3-Methyladipic acid	7.06	[M-H]-	C7H12O4	159.06628	115.07655	97.06592	141.05597
19	3-Methylglutaric/adipic acid	7.15	[M-H]-	C6H10O4	145.05063	101.06105	127.04047	83.05048
20	beta-Hydroxybutyric acid	6.20	[M-H]-	C4H8O3	103.04007	59.01396	57.03466	85.02970
21	Citric acid	7.54	[M-H]-	C6H8O7	191.01973	111.00918	87.00906	129.01990
22	Myristic acid	1.29	[M-H]-	C14H28O2	227.20166	-	-	-
23	Palmitic acid	1.32	[M-H]-	C16H32O2	255.23296	-	-	-
24	Uric acid	6.96	[M-H]-	C5H4N4O3	167.02106	124.01566	152.01250	-
25	Choline	6.14	[M]+	C5H14NO	104.10699	60.08150	-	-
26	Isobutyrylglycine	6.02	[M-H]-	C6H11NO3	144.06662	74.02489	100.07691	-
27	Proline	6.78	[M+H]+	C5H9NO2	116.07059	70.06561	-	-
28	***p*-fluoro-DL-phenylalanine (IS)**	6.10	[M-H]-	C9H10FNO2	182.06228	165.03494	121.04539	72.00879
29	**U13C D-glucose (IS)**	6.70	[M-H]-	[13]C6H12O6	185.07624	-	-	-

Compounds were identified using authentic standards (level 1 of identification); Compounds in bold represent Internal Standards (IS) [ref. 97].

Supplementary Table S5: List of the target compounds monitored in the method at pH 3.

Nº	Compound name	Rt (min)	Adduct	Molecular formula	Precursor ion	Fragment ion 1	Fragment ion 2	Fragment ion 3
1	L-Tryptophan	6.34	[M+H]+	C11H12N2O2	205.09714	188.07021	146.05972	159.09138
2	L-Histidine	8.00	[M+H]+	C6H9N3O2	156.07674	110.07137	95.06063	-
3	Uridine	4.73	[M-H]-	C9H12N2O6	243.06225	110.02528	200.05753	152.03604
4	Creatine	6.62	[M+H]+	C4H9N3O2	132.07675	90.05546	-	-
5	Glutamic acid	7.10	[M+H]+	C5H9NO4	148.06042	84.04470	102.05511	130.04967
6	L-Aspartic acid	7.19	[M+H]+	C4H7NO4	134.04477	74.02417	88.03965	116.03428
7	L-Serine	7.30	[M+H]+	C3H7NO3	106.04986	60.04494	88.03959	70.02920
8	L-Alanine	6.90	[M+H]+	C3H7NO2	90.05495	-	-	-
9	Creatinine	6.47	[M+H]+	C4H7N3O	114.06619	86.07156	72.04483	-
10	L-Arginine	8.00	[M+H]+	C6H14N4O2	175.11894	70.06556	116.07057	130.09727
11	gamma Aminobutyric acid	6.55	[M+H]+	C4H9NO2	104.07061	87.04424	86.06023	69.03388
12	L-Methionine	6.42	[M+H]+	C5H11NO2S	150.05831	104.05294	133.03157	56.05007
13	Ornithine	8.00	[M+H]+	C5H12N2O2	133.09715	70.06579	116.07086	104.96355
14	trans-4-Hydroxy-L-proline	6.87	[M+H]+	C5H9NO3	132.06551	86.06033	68.04995	114.05487
15	Valine	6.47	[M+H]+	C5H11NO2	118.08624	72.08121	55.05482	-
16	L-Threonine	7.08	[M+H]+	C4H9NO3	120.06551	74.06054	56.05013	102.05518
17	L-Cystine	8.00	[M+H]+	C6H12N2O4S2	241.03111	151.98328	120.01146	195.02599
18	Glutamine	7.30	[M+H]+	C5H10N2O3	147.07641	130.04968	84.04470	101.07114
19	Glycine	7.10	[M+H]+	C2H5NO2	76.03930	58.06564	-	-
20	L-Asparagine	7.35	[M+H]+	C4H8N2O3	133.06076	74.02417	87.05571	116.03421
21	Lysine	8.00	[M+H]+	C6H14N2O2	147.11279	84.08112	130.08611	-
22	Tyrosine	6.54	[M+H]+	C9H11NO3	182.08116	136.07547	165.05432	123.04399
23	***p*-fluoro-DL-phenylalanine (IS)**	6.10	[M+H]+	C9H10FNO2	184.07683	138.07109	118.06506	149.03932
24	**U13C L-Glutamine (IS)**	7.14	[M+H]+	[13]C5H10N2O3	152.09319	135.06660	105.08461	-

Compounds were identified using authentic standards (level 1 of identification); Compounds in bold represent Internal Standards (IS) [ref. 97].

Supplementary Table S6: Assigned metabolites with significant differences among groups according to the non-targeted MS-metabolomics in the negative mode.

m/z	Rt	Adduct	Molecular formula	Error (ppm)	Description	CV (%) in QC
135.0305	5.26	[M-H]-	C5H4N4O	5.39	Hypoxanthine[#1]	23
117.0187	6.84	[M-H]-	C4H6O4	5.35	methylmalonic acid[#1]	17
130.0509	6.41	[M-H]-	C5H9NO3	0.25	4-Hydroxyproline[#1]	26
177.0409	6.76	[M-H2O-H]-	C6H12O7	2.23	D-Gluconic acid[#2]	7
146.0454	6.69	[M-H]-	C5H9NO4	2.59	N-acetyl-serine[#2]	28
103.0404	6.21	[M-H]-	C4H8O3	3.19	beta Hydroxybutyrate[#1]	14
112.0510	5.31	[M-H]-	C4H7N3O	4.73	Creatinine[#1]	14
104.0348	7.33	[M-H]-	C3H7NO3	3.96	Serine[#1]	24
279.2331	1.27	[M-H]-	C18H32O2	0.52	9Z,12Z-Linoleic acid[#2]	15
303.2341	1.25	[M-H]-	C20H32O2	3.49	8,11-eicosadienoic acid[#2]	25
102.0560	6.88	[M-H]-	C4H9NO2	0.46	3-Aminobutyric acid[#2]	14

#n—indicates the metabolite level identification according to the Metabolomics Standards Initiative (MSI) [ref. 97].

Supplementary Table S7: Assigned metabolites according to the non-targeted MS-metabolomics in the positive mode.

m/z	Rt	Adduct	Molecular formula	Error (ppm)	Description	CV (%) in QC
114.0667	5.66	[M+H]+	C4H7N3O	4.21	Creatinine[#1]	27
137.0457	4.89	[M+H]+	C5H4N4O	1.02	Hypoxanthine[#1]	12
205.0983	6.08	[M+H]+	C11H12N2O2	5.75	Tryptophan[#1]	8
90.0558	6.76	[M+H]+	C3H7NO2	8.99	Alanine[#1]	10
156.0774	7.87	[M+H]+	C6H9N3O2	4.10	Histidine[#1]	4
205.0974	1.44	[M+H]+	-	-	Indolyl carboxylic acids and derivatives[#3]	9
118.0868	5.88	[M+H]+	C5H11NO2	4.66	Betaine[#2]	27
106.0505	7.18	[M+H]+	C3H7NO3	6.32	Serine[#1]	14
258.1101	7.17	[M+H]+	C8H20NO6P	0.04	Glycerophosphocholine[#2]	22
153.0412	5.37	[M+H]+	C5H4N4O2	3.46	Xanthine[#1]	6
120.0657	2.97	[M+H]+	C4H9NO3	1.75	L-Homoserine/2-methylserine/4-Amino-3-hydroxybutyrate[#2]	14
203.1513	7.58	[M+H]+	C8H18N4O2	4.92	Asymmetric dimethylarginine[#1]	9
248.1513	5.87	[M+H]+	C11H21NO5	8.22	3-Hydroxybutyrylcarnitine[#2]	18
175.1196	7.87	[M+H]+	C6H14N4O2	3.71	Arginine[#1]	8
76.0402	6.97	[M+H]+	C2H5NO2	11.18	Glycine[#1]	17
279.2325	1.19	[M+H-H2O]+	C18H32O3	2.22	9(10)-Epoxy-12Z-octadecenoic acid[#2]	6
205.1560	7.76	[M]+	C9H21N2O3	6.29	3-Hydroxy-N6,N6,N6-trimethyl-L-lysine[#2]	10
162.1129	6.37	[M+H]+	C7H15NO3	2.78	Carnitine[#2]	2

#n—indicates the metabolite level identification according to the Metabolomics Standards Initiative (MSI) [ref. 97].

Supplementary Figure S1

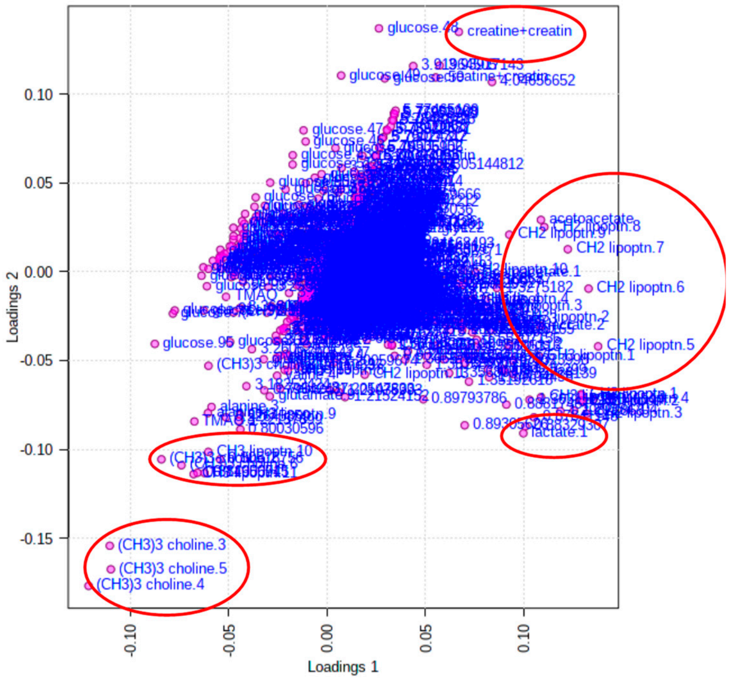

Supplementary Figure S1. PCA loading factors plot highlights the discriminant metabolites for the separation of groups according to ^{1}H NMR-based metabolomics. Metabolites circle in red, $(CH_3)_3$ choline, creatine/creatinine, lactate, acetoacetate, CH_2 and CH_3 lipoproteins contributed most to discriminating severe COVID-19 patients from controls.

Supplementary Figure S2

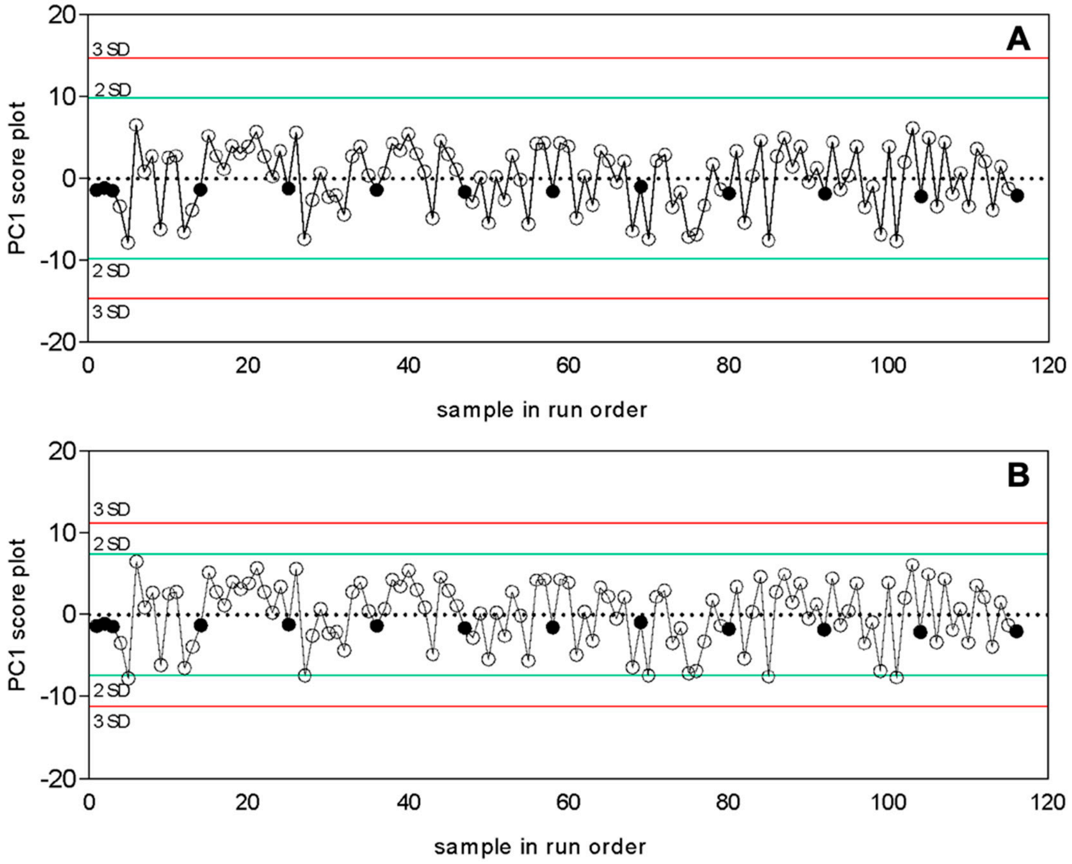

Supplementary Figure S2. PC1 scores plot versus sample in run order at (A) pH 8 and (B) pH 3 analyses indicating the stability of the system throughout the analytical run. The black dots represent the QC scores and the white dots represent specimen samples. The figure also illustrates the 2 and 3 standard deviation limits which are shown as solid lines colored in green and red, respectively.

Supplementary Figure S3

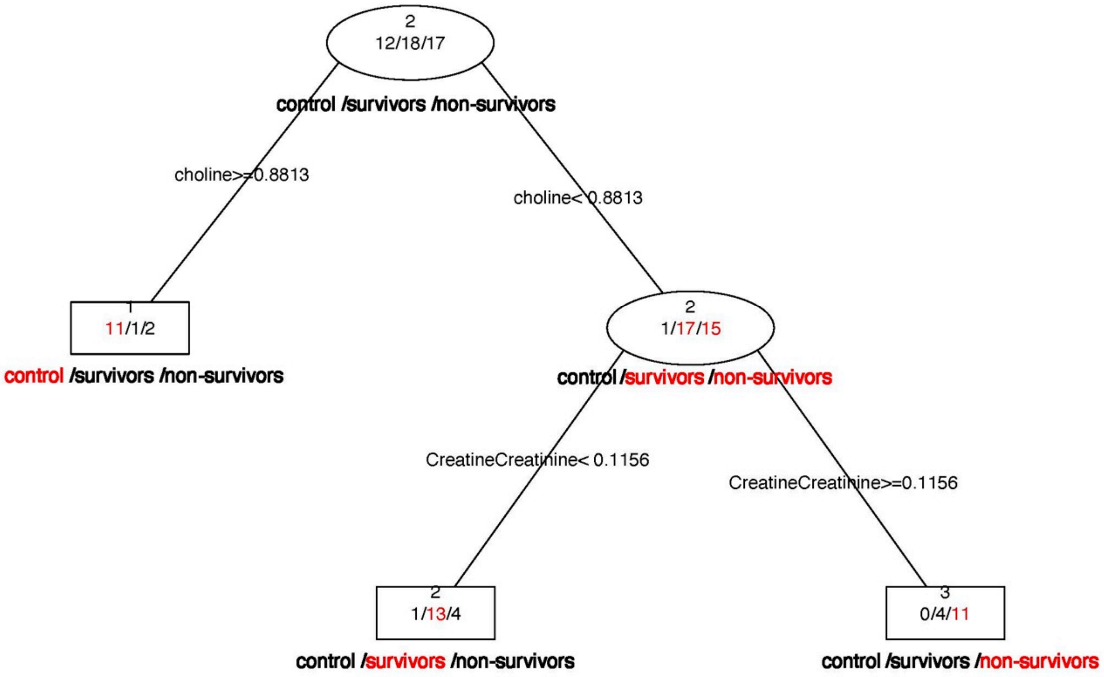

Supplementary Figure S3. Classification and Regression Tree (CART) model indicates that $(CH_3)_3$-choline related metabolites and creatine/creatinine present high predictive power in assigning subjects according to their morbidity class.

The CART model was built using the metabolites that were found to be discriminatory in the multivariate PCA analysis, which was based on ^{1}H NMR metabolomics: $(CH_3)_3$-choline-related metabolites, creatine/creatinine, glutamine, alanine, histidine, tyrosine, valine, leucine, isoleucine, CH_2 of lipoproteins (VLDL), CH_3 of lipoproteins, CH=CH olefinic protons of triacylglycerols, acetoacetate, lactate, acetate, formate, *N*-acetyl of glycoproteins. Sex and age of the subjects were also included in the model. In red is the classification that best predicted the morbidity class for control subjects (partition point choline ≥ 0.8813), survivors -creatine/creatinine < 0.1156 and non-survivors -creatine/creatinine ≥ 0.1156.

Supplementary Figure S4

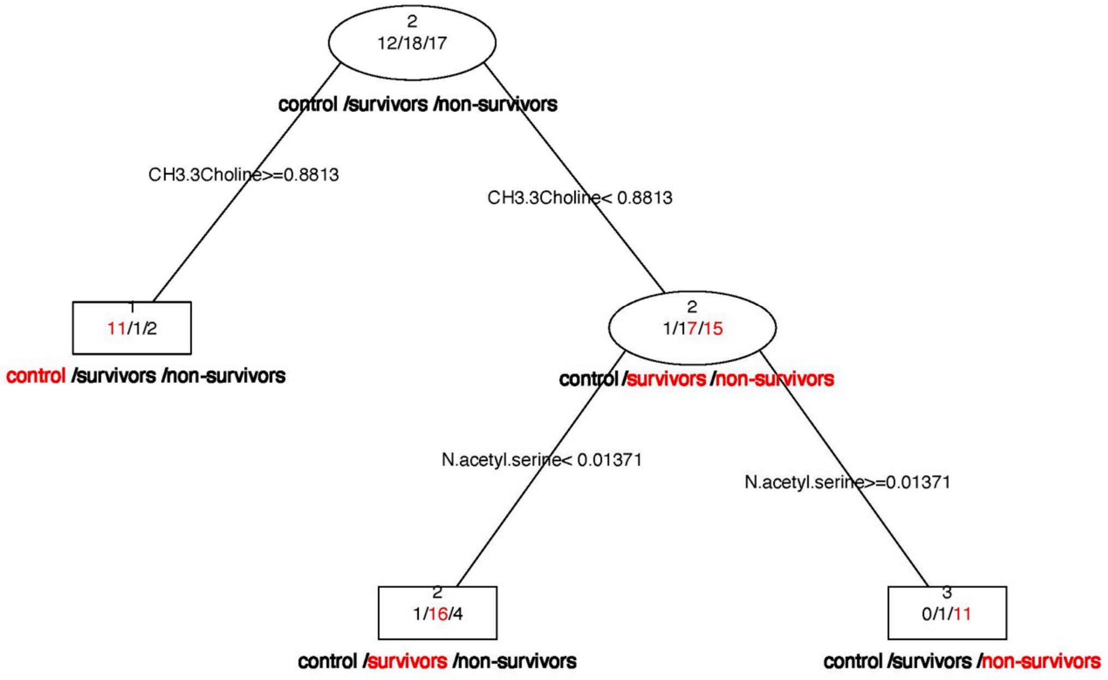

Supplementary Figure S4. Classification and Regression Tree (CART) model indicates that CH_3-choline related metabolites and *N*-acetylserine present high predictive power in assigning subjects to their morbidity class.

The CART model was built using the metabolites that were found to be discriminatory in the NMR and MS- analysis: $(CH_3)_3$-choline-related metabolites, creatine/creatinine, glutamine, alanine, histidine, tyrosine, valine, leucine, isoleucine, serine, betaine, hypoxanthine, xanthine, tryptophan, 4-hydroxyproline, gluconic acid, *N*-acetylserine, asymmetric dimethylarginine, methylmalonic acid, β-hydroxybutyrate, CH_2 of lipoproteins (VLDL), CH_3 of lipoproteins, CH=CH olefinic protons of triacylglycerols, acetoacetate, lactate, acetate, formate, *N*-acetyl of glycoproteins. Sex and age of the subjects were also included in the model. In red is the classification that best predicted the morbidity class for control subjects (partition point choline ≥0.8813), survivors - *N*-acetylserine < 0.0137 and non-survivors - *N*-acetylserine ≥ 0.0137.

Supplementary Figure S5

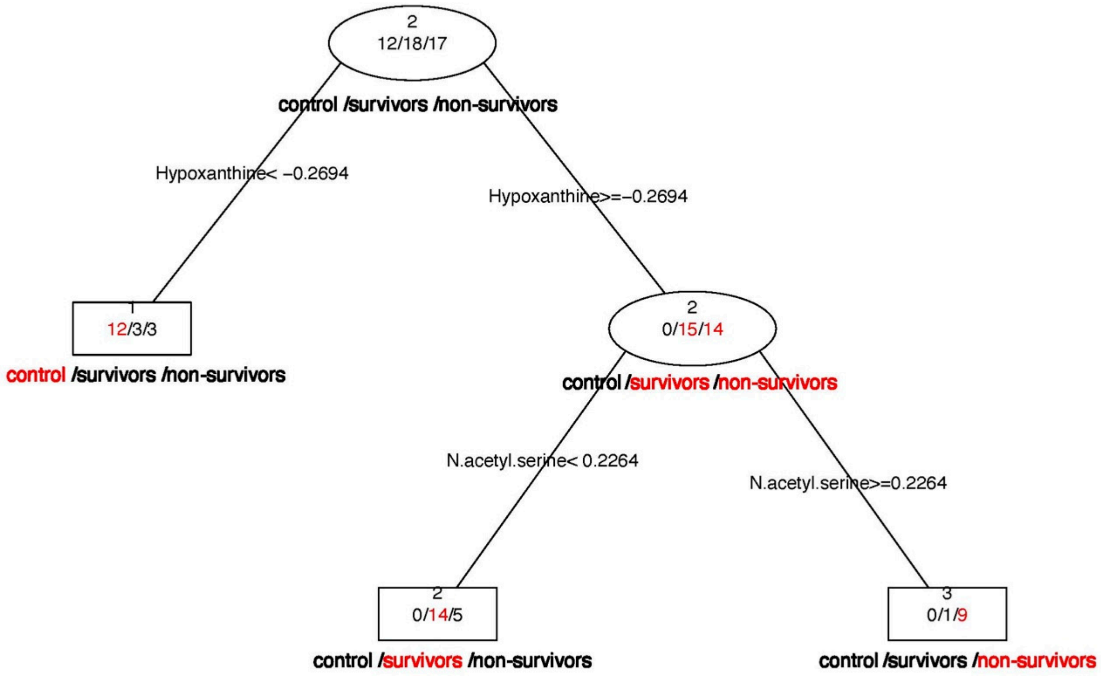

Supplementary Figure S5. Classification and Regression Tree (CART) model indicates that hypoxanthine and *N*-acetylserine present high predictive power in assigning subjects to their morbidity class.

The CART model was built using the metabolites that were found to be discriminatory in the MS analysis: glycerophosphocholine, serine, betaine, histidine, alanine, hypoxanthine, xanthine, creatinine, 4-hydroxyproline, gluconic acid, *N*-acetylserine, asymmetric dimethylarginine, methylmalonic acid, β-hydroxybutyrate, tryptophan. Sex and age of the subjects were also included in the model. In red is the classification that best predicted the morbidity class for control subjects (partition point choline < -0.2694), survivors *N*-acetylserine < 0.2264 and non-survivors - *N*-acetylserine >= 0.1156.

Supplementary Figure S6

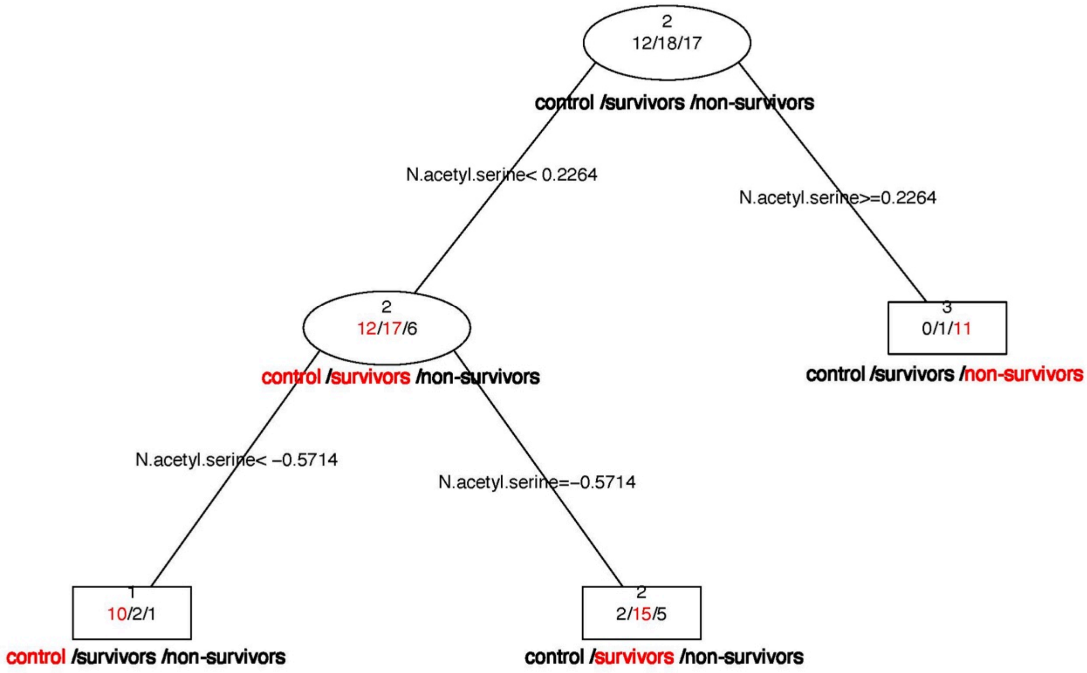

Supplementary Figure S6. Classification and Regression Tree (CART) model indicates that *N*-acetylserine presents high predictive power in assigning subjects to their morbidity class.

The CART model was built using the metabolites that were found to be higher in the non-survivors compared to survivors and control subjects: *c*reatinine, 4-hydroxyproline, gluconic acid and *N*-acetylserine. Sex and age of the subjects were also included in the model. In red is the classification that best predicted the morbidity class with the partition point *N*-acetylserine < 0.2264 for control and survivors subjects and >= 0.2264 for non-survivors.

References

1. Sumner, L.W.; Amberg, A.; Barrett, D.; Beale, M.H.; Beger, R.; Daykin, C.A.; Fan, T.W.-M.; Fiehn, O.; Goodacre, R.; Griffin, J.L.; et al. Proposed minimum reporting standards for chemical analysis Chemical Analysis Working Group (CAWG) Metabolomics Standards Initiative (MSI). *Metabolomics* **2007**, *3*, 211–221. https://doi.org/10.1007/s11306-007-0082-2 [ref. 97]
